# Supplementary material for: A single full-length VAR2CSA ectodomain variant purifies broadly neutralizing antibodies against placental malaria isolates
Source: eLife. 2022 Feb 1;11:e76264. doi: 10.7554/eLife.76264 (PMC8959597; doi:10.7554/eLife.76264)
Supplement: Supplementary file 1. [file elife-76264-supp1.docx]

**Table S1**

|  | Binding to CSA (Average number of bound IE per mm2) |
| --- | --- |
| NF54 | 236.2 |
| FCR3 | 507.9 |
| M. Camp | 148.2 |
| M0736 | 221.5 |
| M2022170 | 61 |
| M2001190 | 591 |
| WF12/7G8 | 864.7 |
